# Hypoxia exposure blunts angiogenic signaling and upregulates the antioxidant system in endothelial cells derived from elephant seals

**DOI:** 10.1186/s12915-024-01892-3

**Published:** 2024-04-23

**Authors:** Kaitlin N. Allen, Julia María Torres-Velarde, Juan Manuel Vazquez, Diana D. Moreno-Santillán, Peter H. Sudmant, José Pablo Vázquez-Medina

**Affiliations:** 1 Department of Integrative Biology, University of California Berkeley, Berkeley, CA 94720 USA; 2 Center for Computational Biology, University of California Berkeley, Berkeley, CA 94720 USA

**Keywords:** Diving, Redox, Marine mammal, Inflammation, Glutathione, Ischemia/reperfusion

## Abstract

**Background:**

Elephant seals exhibit extreme hypoxemic tolerance derived from repetitive hypoxia/reoxygenation episodes they experience during diving bouts. Real-time assessment of the molecular changes underlying protection against hypoxic injury in seals remains restricted by their at-sea inaccessibility. Hence, we developed a proliferative arterial endothelial cell culture model from elephant seals and used RNA-seq, functional assays, and confocal microscopy to assess the molecular response to prolonged hypoxia.

**Results:**

Seal and human endothelial cells exposed to 1% O_2_ for up to 6 h respond differently to acute and prolonged hypoxia. Seal cells decouple stabilization of the hypoxia-sensitive transcriptional regulator HIF-1α from angiogenic signaling. Rapid upregulation of genes involved in glutathione (GSH) metabolism supports the maintenance of GSH pools, and intracellular succinate increases in seal but not human cells. High maximal and spare respiratory capacity in seal cells after hypoxia exposure occurs in concert with increasing mitochondrial branch length and independent from major changes in extracellular acidification rate, suggesting that seal cells recover oxidative metabolism without significant glycolytic dependency after hypoxia exposure.

**Conclusions:**

We found that the glutathione antioxidant system is upregulated in seal endothelial cells during hypoxia, while this system remains static in comparable human cells. Furthermore, we found that in contrast to human cells, hypoxia exposure rapidly activates HIF-1 in seal cells, but this response is decoupled from the canonical angiogenesis pathway. These results highlight the unique mechanisms that confer extraordinary tolerance to limited oxygen availability in a champion diving mammal.

**Supplementary Information:**

The online version contains supplementary material available at 10.1186/s12915-024-01892-3.

## Background

Repeated bouts of extreme hypoxemia followed by reoxygenation characterize diving in northern elephant seals (*Mirounga angustirostris*) [1–4]. In the vasculature, hypoxia exposure shifts the endothelium toward a pro-inflammatory state [5–7], priming vessels for increased leukocyte adhesion upon reoxygenation [8]. In addition, hypoxic changes in mitochondrial metabolism lead to succinate accumulation and subsequent superoxide generation via reverse electron transport [9–11], though the degree of succinate accumulation may modulate these effects [12]. Reoxygenation reintroduces oxygen to the “primed” endothelium, driving additional oxidant generation from various sources including NADPH oxidases and the electron transport chain. The resulting oxidative damage promotes endothelial dysfunction, which compromises both the barrier and vasoregulatory roles of the endothelial layer [13].

Hypoxia-induced endothelial dysfunction would catastrophically impact the dive response in marine mammals, for whom persistent peripheral vasoconstriction and fine-scale modulation of vascular tone are critical during prolonged submersion [14–16]. Recent work shows that nitric oxide responsiveness differs between organs and their supplying arteries in Weddell seals [15] and that inflammatory signaling is blunted in Weddell and northern elephant seal blood [17], suggesting that diving mammals tightly regulate the inflammatory response to mitigate vascular damage during diving. Additionally, marine mammals exhibit a robust antioxidant defense system which likely counteracts the damaging effects of diving-induced oxidant generation. In particular, marine mammals possess high tissue and circulating levels of glutathione (GSH), the most abundant cellular thiol and a crucial cofactor for several antioxidant enzymes [18–23].

Besides comparative biochemical studies showing that marine mammals exhibit high antioxidant levels, recent work shows duplication events and positive selection on candidate genes involved in GSH metabolism in diving mammals [24–26], but the dynamic regulation of the antioxidant system during hypoxia and diving remains unknown. Comprehensive assessments of antioxidant function and immunomodulation remain infeasible during diving due to technological limitations on in vivo studies and the inaccessibility of the animals when diving at sea. To this end, we developed a primary cell culture system using arterial endothelial cells derived from expelled placentas obtained during elephant seal breeding haulouts. Arteries are generally inaccessible in living marine mammals, and sampling of arterial tissue is constrained to expelled tissues or those derived from necropsy. We then conducted comparative transcriptomic and metabolic analyses to study the molecular mechanisms that drive hypoxemic tolerance in seal endothelial cells. We found that rapid hypoxic stabilization of HIF-1α in elephant seal cells co-occurred with downregulated inflammatory signaling and a highly dynamic transcriptional response promoting vascular homeostasis alongside a blunted migratory response to hypoxia. Moreover, hypoxia increased GSH and succinate levels in seal but not human cells in coordination with enhanced respiratory capacity following hypoxia/reoxygenation. Together, these results highlight the importance of the GSH antioxidant system and mitochondrial function in protecting the vascular endothelium in a champion diving mammal exposed to extreme oxygen fluctuations**.**

## Results

### Characterization of elephant seal arterial endothelial cells in primary culture

**Fig. 1:**
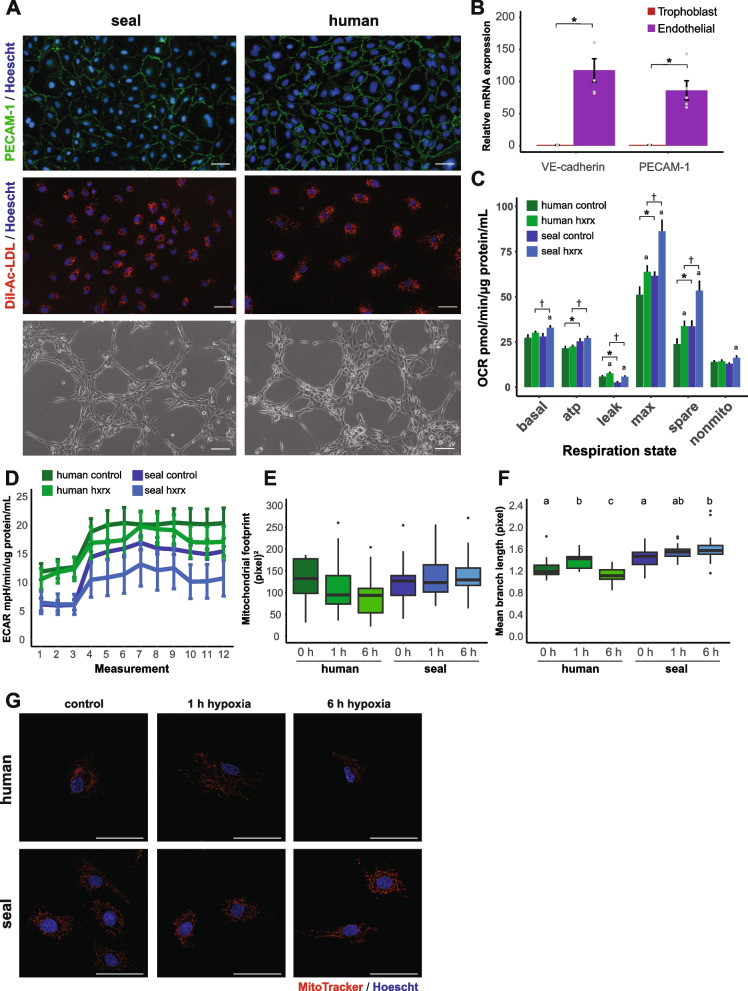
Primary elephant seal arterial endothelial cells express canonical endothelial markers and modulate cellular respiration in response to hypoxia/reoxygenation. **A** Top: PECAM-1 (CD31; green) staining in seal and human endothelial cells. Middle: DiI-acetylated LDL (red) uptake by seal and human cells. Nuclei (blue) in the top and middle panels are stained with Hoechst 33342. Lower: Seal and human endothelial cells spontaneously form tubes when cultured in a 3D gel matrix. **B** Relative mRNA expression levels of the endothelial markers VE-cadherin (CD144) and PECAM-1 (CD31) in seal endothelial (purple) compared to placental trophoblast (red) cells, *n* = 3–5 replicates per group. Individual points are shown as white circles with gray borders. *p < 0.05. **C** Mitochondrial function at baseline and after hypoxia/reoxygenation in seal and human cells. Values are normalized to protein content per well. hxrx, hypoxia/reoxygenation. *p < 0.05 between species at baseline. †*p* < 0.05 between species after hypoxia/reoxygenation. Lettering indicates intraspecific changes between baseline and hypoxia/reoxygenation, *n* = 9 per group. **D** Extracellular acidification rate (ECAR) in seal and human endothelial cells at baseline and after hypoxia/reoxygenation, *n* = 9 per group. **E, F** Mitochondrial footprint (**E**) and mean mitochondrial network branch length (**F**) in seal and human endothelial cells after hypoxia/reoxygenation. Hypoxia exposure occurred for 1 or 6 h, followed by 30 min reoxygenation. Lettering indicates intraspecific changes between baseline and hypoxia/reoxygenation, *n* = 13–28 cells per group. **G** Representative images of human (top) and seal (bottom) endothelial cells stained with MitoTracker Red CMXRos after 0, 1, and 6 h hypoxia followed by 30 min reoxygenation. See Additional file 1: Fig. S1 for additional representative images. Scale bar in all images is 50 µm

We generated a novel cellular model to study the molecular response to hypoxia in elephant seals. Primary endothelial cells derived from seal and human placental arteries stain positive for the endothelial marker PECAM-1 (CD31), take up acetylated low-density lipoprotein (Ac-LDL), and spontaneously form tubes when cultured on a 3D matrix (Fig. 1A). Moreover, CD144 (VE-cadherin) and CD31 (PECAM-1) mRNA expression is enriched in seal endothelial cells compared to placental trophoblast cells (CD144: 117-fold, *t* = 6.501, *p* = 0.003; CD31: 86-fold, *t* = 5.665, *p* = 0.005) (Fig. 1B). These results support an endothelial lineage for the isolated cell cultures.

Basal oxygen consumption rates do not differ between seal and human cells, though exposure to hypoxia/reoxygenation increased basal oxygen consumption only in seal cells. Furthermore, ATP-linked respiration, maximal respiration, and spare respiratory capacity were greater in seal than in human cells at baseline and after hypoxia/reoxygenation despite hypoxia/reoxygenation-induced increases in maximal respiration and spare capacity in both species (Fig. 1C). In contrast, human cells displayed greater proton leak than seal cells both at baseline and after hypoxia/reoxygenation, though leak increased in both species following hypoxia/reoxygenation (Fig. 1C). While seal cells displayed the highest overall oxygen consumption rates after hypoxia/reoxygenation (Fig. 1C), these cells also exhibited the lowest extracellular acidification rates, suggesting that glycolytic acidification of the media is not a primary component of the increased respiratory capacity (Fig. 1D).

We then assessed mitochondrial networks in seal and human cells using confocal microscopy to determine whether the observed differential changes in oxygen consumption may be driven by morphological differences in mitochondria following hypoxia/reoxygenation. Mitochondrial footprint appeared to decline in human cells after 1 and 6 h hypoxia followed by 30 min reoxygenation, though this trend was not statistically significant (Fig. 1E,G; Additional file 1: Fig. S1). In contrast, mitochondrial branch length increased transiently in human cells after 1 h hypoxia but dropped below baseline levels by 6 h (*χ*^2^(3) = 17.47, *p* = 0.0002; Fig. 1E). In seal cells, however, hypoxia increased branch length from control to 6 h (*χ*^2^(3) = 9.000, *p* = 0.01; Fig. 1F). These results suggest that seal cells increase mitochondrial connectivity while maintaining overall volume during hypoxia in contrast to long-term decreases in branching and volume in human cells.

### Hypoxia exposure differentially regulates HIF-1 signaling in seal and human cells

**Fig. 2:**
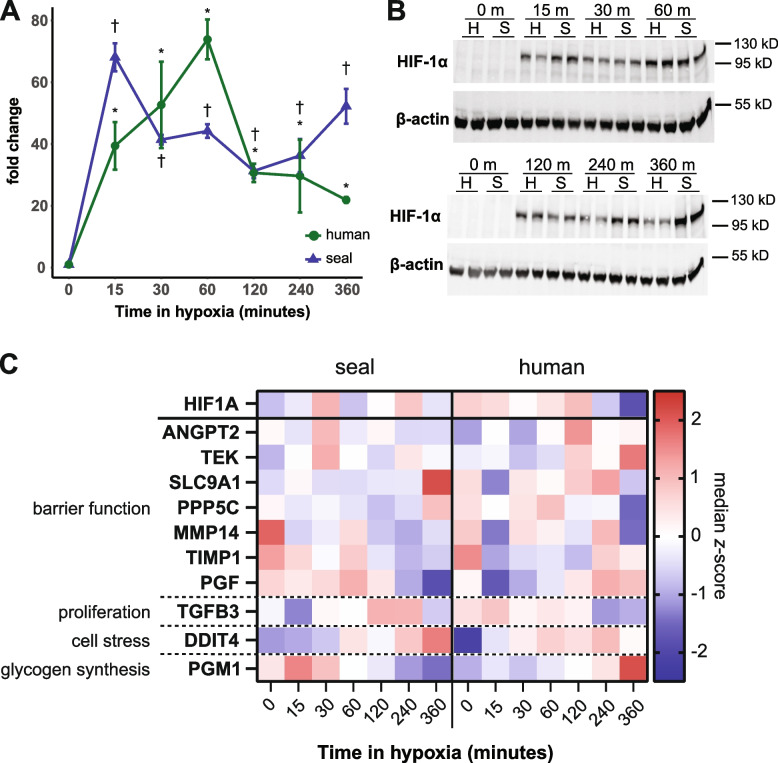
Hypoxia rapidly stabilizes HIF-1α in seal cells. **A** Fold change in HIF-1α protein abundance compared to normoxic baseline in endothelial cells exposed to 1% O_2_ for up to 6 h, *n* = 3. †*p* < 0.05 versus control (seal); *p < 0.05 versus control (human). **B** Representative western blots showing HIF-1α abundance. H human, S seal. Full images of gels are available in Additional file 1: Fig. S2. All samples derive from the same experiment and were distributed across four gels in total, with inter-gel controls loaded on each gel. All gels were processed in parallel. **C** Median mRNA *z*-scores for HIF-1α and select HIF-1 target genes in seal and human cells

We evaluated the HIF-1-mediated response to sustained hypoxia in seal and human cells. Both human and seal cells remained viable (> 99%) throughout a 6 h exposure to 1% O_2_ (Additional file 2: Table S1). Hypoxia increased HIF-1α protein levels to a maximum of ~ 70-fold over (normoxic) baseline abundance in both species (*p* = 0.005 for both species) (Fig. 2A,B; Additional file 1: Fig. S2), though we observed species-specific dynamics. HIF-1α abundance was biphasic in seal cells: an initial major peak within 15 min of hypoxia was followed by a ~ 45% decline from 30 to 120 min, with levels again increasing after 2 h. This late-stage increase co-occurred with an increase in HIF-1α mRNA levels (Fig. 2C). In contrast, HIF-1α protein levels did not peak in human cells until 1 h in hypoxia, and HIF-1α abundance declined continuously following this peak. HIF-1α mRNA levels in human cells remained relatively stable through 2 h, followed by a precipitous decline at later timepoints (Fig. 2C). Together, these data suggest that hypoxic HIF-1α stabilization is generally conserved despite the observed variations in the dynamics of the response between species.

Seal and human cells displayed distinct gene expression patterns for several known HIF-1 targets (Fig. 2C). Expression of factors implicated in extracellular matrix reorganization, angiogenesis, and cell motility were elevated across the time course in human cells but were generally repressed or lagged behind in seal cells, including angiopoietin 2 (ANGPT2), the angiopoietin 1 receptor (TEK; TIE2), the Na+ /H+ antiporter (SLC9A1; NHE1), protein phosphatase 5 catalytic subunit (PPP5C), transforming growth factor beta-3 (TGFB3), and matrix metalloprotease 14 (MMP14), while a metalloprotease inhibitor (TIMP1) showed the opposite expression pattern (Fig. 2C). Placental growth factor (PGF) was downregulated in seal cells after 2-h hypoxia but displayed the opposite trend in human cells. Expression of the cellular stress protein DNA damage-inducible transcript 4 (DDIT4; REDD1) increased in seal cells only at late timepoints, but its expression lagged that of human cells (Fig. 2C). Moreover, seal cells increased expression of the first enzyme in glycogen synthesis, phosphoglucomutase 1 (PGM1), at early timepoints while PGM1 was generally repressed in human cells (Fig. 2C). Together, these results suggest that angiogenic signaling is decoupled from HIF-1α stabilization in seal cells exposed to hypoxia.

### Seal cells delay the onset of pro-angiogenic transcriptional programs during hypoxia

**Fig. 3:**
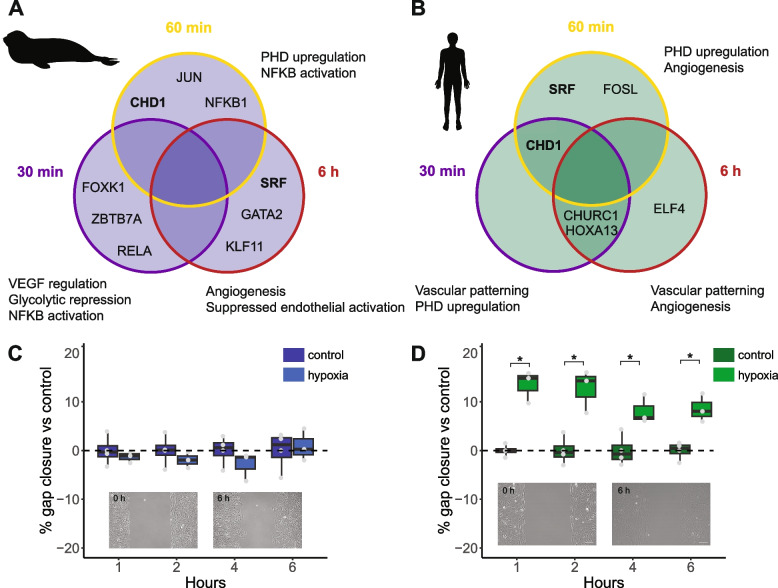
Seal cells delay angiogenic signaling and do not increase cell migration in response to hypoxia exposure. **A**, **B** Top three transcription factors predicted from genes DE at 30 min, 60 min, or 6 h versus control for (**A**) seal and (**B**) human cells. Bold text indicates factors shared between species. **C**, **D** Rates of gap closure in (**C**) seal and (**D**) human endothelial cells in hypoxia compared to control. Data are normalized to normoxic controls at respective time points to account for overall cell migration over time. Data are *n* = 4 from two independent experiments. In the boxplots, lower and upper hinges correspond to the first and third quartiles. Whiskers are 1.5*IQR. Individual points are shown as gray circles. *p < 0.05 versus control. Inset: representative images of scratch at 0 h and after 6 h cell migration. Scale bar is 50 µm. Raw gap closure rates are available in Additional file 1: Fig. S3

We next used RNAseq to evaluate global changes in gene expression in seal and human cells during a 6-h hypoxia exposure. We conducted *cis-*regulatory analyses with differentially expressed (DE) genes to predict transcription factors (TFs) that play major roles in the response to hypoxia at each time point. Comparative analyses of the top three predicted TFs in seal and human cells after 30 min, 60 min, and 6 h in hypoxia revealed species-specific dynamics in transcriptional control: none of the nine predicted seal TFs overlapped across time points, while only six distinct TFs were predicted in human cells (Fig. 3A,B). Two TFs (chromodomain helicase DNA-binding protein 1, CHD1; serum response factor, SRF) were represented in both human and seal datasets. Predicted TFs in seal cells at 30 and 60 min suggest rapid metabolic (forkhead box K1, FOXK1) and inflammation-related (NF-κB subunit RELA; NFKB1; JUN; zinc finger and BTB containing 7A, ZBTB7A) responses to hypoxia, with a limited angiogenic signature (JUN). In contrast, the transcriptional signature in human cells at both 30 and 60 min suggests altered expression of oxygen delivery pathways via homeobox A13 (HOXA13), FOS like 1 (FOSL) and SRF, all of which regulate angiogenesis, vascular remodeling, and vasodilation [27–30]. In addition, broad changes in DNA replication and transcription may characterize the human endothelial cell response to hypoxia via churchill domain containing 1 (CHURC1) and CHD1.

Seal cells do appear to regulate the expression of genes involved in oxygen delivery during hypoxia at 6 h. Two of the top three predicted TFs in seal cells at 6 h (GATA binding protein 2, GATA2; SRF) regulate angiogenesis, vascular remodeling, and vascular tone [27, 31]. The third TF, Kruppel-like factor 11 (KLF11) maintains vascular homeostasis and limits endothelial oxidant stress and inflammation via inhibition of NADPH oxidase 2 (NOX2) and matrix metalloproteinase 9 (MMP9) expression [32, 33]. In human cells, repeated identification of HOXA13 and CHURC1 suggests a continued angiogenic signal at 6 h along with upregulation of transcription. Additionally, ELF4-mediated transcriptional changes may promote cell cycle entry during hypoxia exposure [34, 35]. Cell migration data support these data, as hypoxia accelerated migration into a cell-free gap in human but not seal cells over a 6-h exposure (Fig. 3C,D; Additional file 1: Fig. S3). Together, these results suggest a highly dynamic transcriptional response to hypoxia in seal cells which alters cellular metabolism to promote endothelial homeostasis. This response stands in contrast to the consistently pro-angiogenic signal observed in human cells exposed to hypoxia.

### The transcriptional response to hypoxia highlights species-specific differences in antioxidant gene expression

**Fig. 4:**
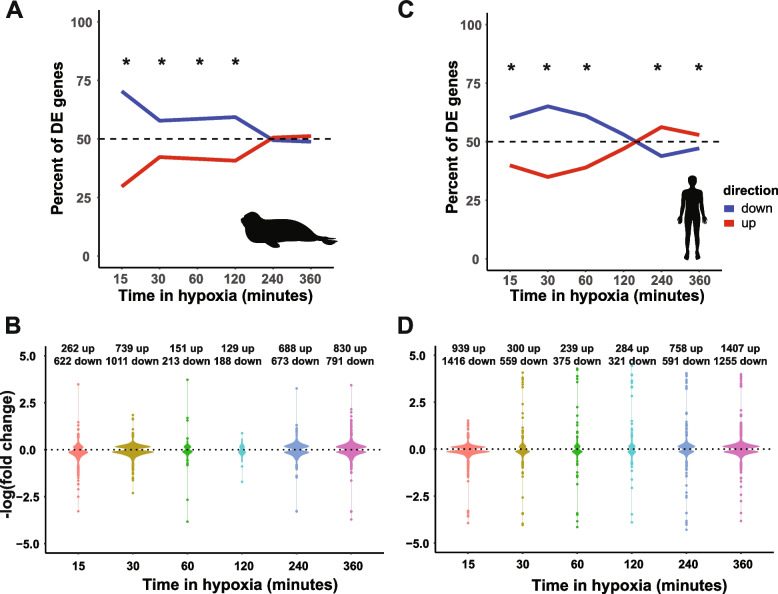
Seal cell gene expression equilibrates under sustained hypoxia exposure. Global differential gene expression patterns in seal (**A**, **B**) and human (**C**, **D**) cells during hypoxia compared to normoxic control. *p < 0.05 compared to 50/50

Hypoxia rapidly repressed global gene expression in both seal and human cells, with 70% of DE genes in seal and 60% of DE genes in human cells downregulated at 15 min compared to control (*p* < 0.001 for both species) (Fig. 4). Gene expression in seal cells remained repressed from 30 min until 4 h (*p* < 0.005 at each time point), at which point up- versus down-regulation did not differ from 50/50 for the remainder of the experiment (4 h: *p* = 0.68; 6 h: *p* = 0.36) (Fig. 4A,B). In human cells, repression was similarly maintained through 1 h (*p* < 0.001 at each time point) and shifted toward upregulation from 4 to 6 h (4 h: *p* < 0.001; 6 h: *p* < 0.005) (Fig. 4C,D).

**Fig. 5:**
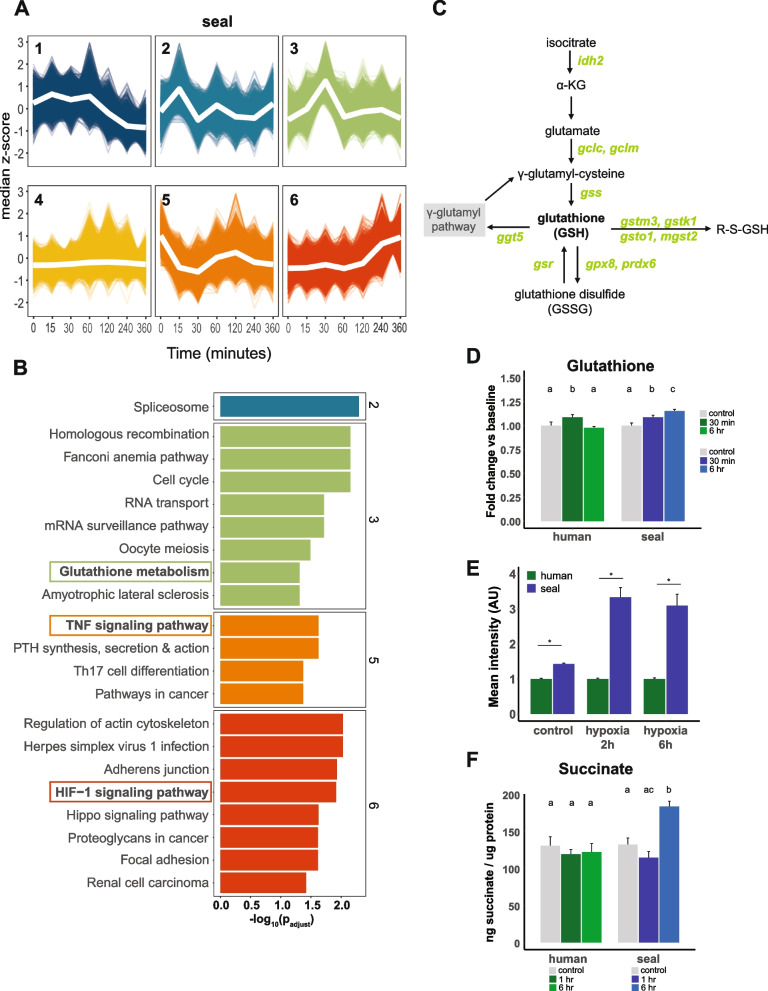
Concerted changes in gene expression in response to hypoxia exposure in seal cells. **A** *k*-means clustering of gene expression data for seal cells exposed to 1% O_2_ for up to 6 h. **B** KEGG pathway enrichment for seal clusters. The numbers on the right axis correspond to seal cluster numbers. See Additional file 1: Fig. S5 for human cell data. **C** Diagrammatic pathway of glutathione synthesis. Green italicized gene symbols represent genes in seal cluster 3. **D** GSH content relative to each species’ baseline. Lettering indicates intraspecific changes among treatments, *n* = 7–8 per group. **E** Mean intensity of intracellular ThiolTracker Violet fluorescence during hypoxia exposure, using *n* = 4 fields per sample and *n* = 3 per group. Data are normalized to human cell values at each time point. *p < 0.05 between species. **F** Percent change in intracellular succinate concentrations in response to hypoxia exposure, *n* = 3 per group. *p < 0.05 versus species baseline

We identified concerted changes in gene expression using *k*-means clustering and conducted gene set enrichment analysis (GSEA) for KEGG pathways within each cluster (Fig. 5A,B; Additional file 1: Fig. S4, Fig. S5). Clustering was performed for *k* = 1…30 clusters; six clusters were selected for final analysis as diminishing returns were observed beyond *k* = 6 (Additional file 1: Fig. S4). Seal cluster 6 and human cluster 3 were both enriched for HIF-1 signaling. Seal cluster 6 contained genes that gradually increased over time, while human cluster 3 genes exhibited a gradual increase in expression until 4 h, followed by a rapid decrease at 6 h. Seal cluster 5 and human cluster 6 were enriched for TNF signaling; both clusters displayed a rapid decrease in expression followed by a gradual return to baseline, suggesting that both human and seal cells downregulate pro-inflammatory pathways during the initial response to hypoxia.

Seal cluster 3 was enriched for glutathione (GSH) metabolism; this cluster contained genes for which expression peaked within 30 min of hypoxia onset. No human gene expression cluster displayed enrichment for genes involved in GSH metabolism (Additional file 1: Fig. S5). Genes from the GSH metabolism pathway present in seal cluster 3 are listed in Table 1 and include the critical GSH biosynthesis genes glutamate–cysteine ligase catalytic (GCLC) and modifier (GCLM) subunits and glutathione synthetase (GSS) (Fig. 5C). GSH levels transiently increased in human cells during hypoxia exposure but continuously increased in seal cells through 6 h in hypoxia (human: *F*_2,20_ = 4.034, *p* = 0.04; seal: *F*_2,21_ = 11.03, *p* = 0.0005) (Fig. 5D). Furthermore, baseline GSH content was ~ 1.4-fold greater in seal compared to human cells (*t* = 13.26, *p* < 0.001) and remained approximately threefold higher in seal than in human cells during hypoxia exposure (2 h: 3.3-fold, *t* = 8.262, *p* = 0.014; 6 h: 3.1-fold, *t* = 6.422, *p* = 0.022) (Fig. 5E), suggesting that early transcriptional changes during hypoxia exposure support increased GSH pools in seal but not human cells.

**Table 1:** Glutathione metabolism genes in seal cluster 3

Gene symbol	Gene name
*idh2*	Isocitrate dehydrogenase (NADP( +)) 2
*gclc*	Glutamate-cysteine ligase catalytic subunit
*gclm*	Glutamate-cysteine ligase modifier subunit
*gss*	Glutathione synthetase
*ggt5*	Gamma-glutamyltransferase 5
*gsr*	Glutathione-disulfide reductase
*pgd*	Phosphogluconate dehydrogenase
*prdx6*	Peroxiredoxin 6
*gpx8*	Glutathione peroxidase 8
*gsto1*	Glutathione *S*-transferase omega 1
*gstm3*	Glutathione *S*-transferase mu 3
*gstk1*	Glutathione *S*-transferase kappa 1
*mgst2*	Microsomal glutathione *S*-transferase 2
*srm*	Spermidine synthase
*sms*	Spermine synthase
*odc1*	Ornithine decarboxylase 1
*rrm1*	Ribonucleotide reductase catalytic subunit M1
*rrm2b*	Ribonucleotide reductase regulatory TP53 inducible subunit M2B

Several genes involved in mitochondrial metabolism and redox homeostasis were also present in seal cluster 3, including ornithine decarboxylase 1 (ODC1), spermidine synthase (SRM), and spermine synthase (SMS) (Table 1) [36–38]. Mitochondrial succinate accumulation during ischemia (and its associated tissue hypoxia) drives superoxide production via reverse electron transport at mitochondrial complex I upon reoxygenation [10, 11]. Hence, we measured intracellular succinate levels during hypoxia exposure. Interestingly, succinate levels increased 40% in seal but not human cells after 6-h hypoxia (seal: *F*_2,6_ = 19.30, *p* = 0.0024; human: *F*_2,6_ = 0.3164, *p* = 0.74) (Fig. 5F); this relatively mild succinate accumulation may support continued oxidative phosphorylation in seal cells after hypoxia/reoxygenation as observed in Fig. 1C. Peroxiredoxin 6 (PRDX6), microsomal glutathione S-transferase 2 (MGST2), and gamma glutamyl transferase 5 (GGT5), which contribute to glutathione-dependent leukotriene synthesis [39, 40] and may promote vasoconstriction in diving mammals [41], were also present in seal cluster 3 (Table 1). Together, these data suggest that in contrast to human cells, seal endothelial cells maintain cellular GSH levels, remain metabolically active, and maintain vasoconstrictive signaling during hypoxia exposure.

### The short-term transcriptional response to hypoxia in seal cells limits inflammatory signaling

**Fig. 6:**
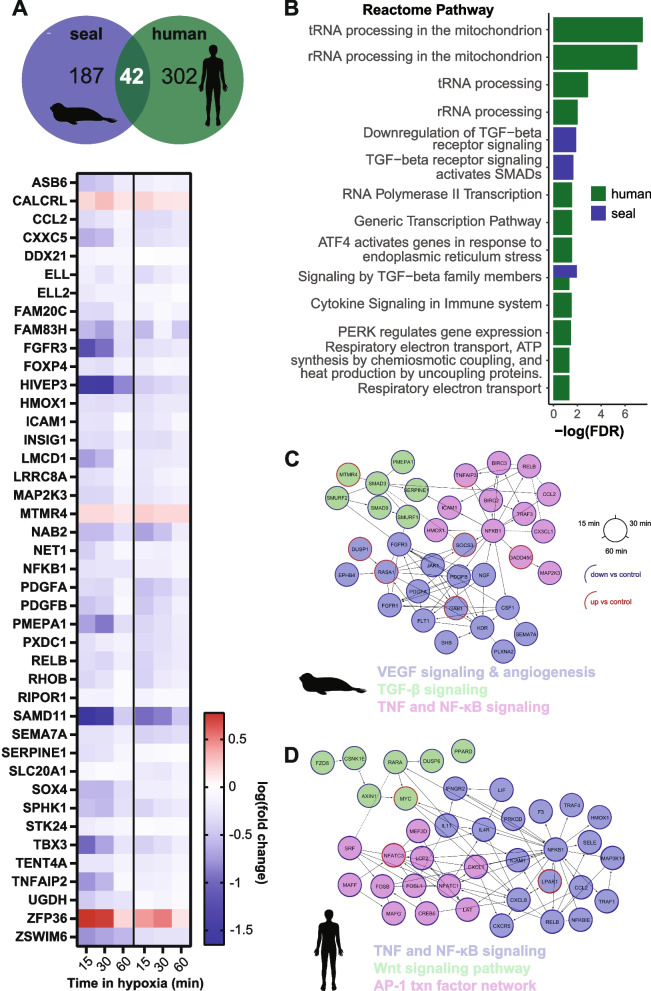
Short-term hypoxia modulates inflammatory signaling in seal cells. **A** Overlap between genes DE at all early time points (15 min, 30 min, 60 min) versus control for seal and human cells. Heatmap shows log_10_(fold change) for 42 DE genes shared between species. **B** Reactome pathway enrichment for genes DE at all early time points. **C**, **D** Functional interaction networks and select enriched pathways for seal (**C**) and human (**D**) genes DE at all early time points

We next investigated whether short-term hypoxia (≤ 1 h) leads to differential functional enrichment in gene expression between species. We detected 42 DE genes shared between the seal and human cell response to short-term hypoxia. Generally, these shared genes displayed similar expression patterns between species (Fig. 6A). Seal genes responsive to short-term hypoxia were enriched for several TGF-β signaling pathways including downregulation of TGF-β receptor signaling. In contrast, the response in human genes was dominated by pathways involved in RNA processing and transcription, ER stress, and upregulation of electron transport chain subunits (Fig. 6B; Additional file 2: Table S2). Functional interaction networks (FINs) suggested a central role for NFKB1 (p50) in the short-term hypoxia response in both species, though expression of this subunit and its related RELB subunit were downregulated relative to control in both species (Fig. 6C,D). The seal FIN also confirmed a critical role for downregulation of TGF-β signaling in seal cells (Fig. 6C) as the only upregulated gene in the network was MTMR4, a TGF-β inhibitor. Interestingly, the angiogenic gene expression signature in seal cells suggested blunting of VEGF-dependent angiogenesis during short-term hypoxia (Fig. 6C).

### Pro-proliferative transcriptional signals characterize the long-term hypoxia response in seal cells

**Fig. 7:**
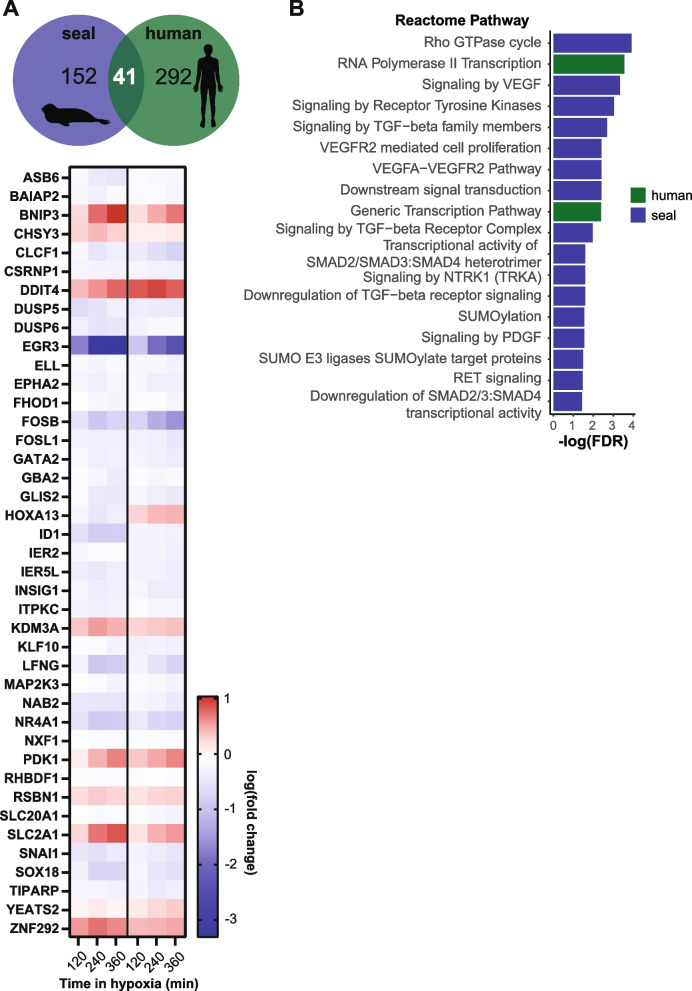
Long-term hypoxia exposure modulates cell proliferation pathways in seal cells. **A** Overlap between genes DE at all late time points (2 h, 4 h, 6 h) versus control for seal and human cells. Heatmap shows log_10_(fold change) for 41 DE genes shared between species. **B** Reactome pathway enrichments for genes DE at 6 h versus control

We next evaluated expression patterns in response to hypoxia exposure for ≥ 2 h. DE genes shared between seal and human cells generally displayed similar trajectories, apart from HOXA13, also predicted as coordinating the human response to hypoxia in cells (Fig. 3B), for which long-term hypoxia repressed expression in seal but stimulated expression in human cells (Fig. 7A). No Reactome pathways were overrepresented in the 193 seal genes DE for 2–6-h hypoxia exposure. Human DE gene expression (333 genes) was again enriched for pathways related to tRNA and rRNA processing and respiratory electron transport (universally upregulated as during short-term exposure) as well as negative regulation of the MAPK pathway (Additional file 1: Fig. S6A; Additional file 2: Table S3).

We then separately considered the transcriptional response to 6-h hypoxia, at which point a second increase in seal HIF-1α protein abundance occurred alongside species-specific expression patterns for several HIF-1 targets (Fig. 2A,C). GSEA revealed downregulation of several pathways related to transcription and mRNA splicing, as well as C-type lectin receptors (implicated in the immune response, cell adhesion, and apoptosis [42]) in human cells at 6 h (Additional file 1: Fig. S6B). Reactome pathway enrichment analyses further showed hypoxia-induced changes in transcription including RNA polymerase II activity in human cells (Fig. 7B). In contrast, seal cell gene expression at 6 h was enriched for several Smad and TGF-β signaling pathways, the VEGFA-VEGFR2 pathway, SUMOylation, and SUMO E3 ligase target proteins (Fig. 7B). Hypoxia-induced suppression of TGF-β signaling appeared to be regulated through increased expression of the Smad phosphatase MTMR4 and the ubiquitin ligase NEDD4L, which targets TGF-β-induced pSmad2/3 (Additional file 2: Table S4) [43, 44]. Despite the observed shift toward pro-proliferative signaling during long-term hypoxia exposure, we did not detect changes in cell migration in seal endothelial cells in response to up to 6-h hypoxia exposure (Fig. 3C,D).

## Discussion

Elephant seals repeatedly experience profound hypoxemia during continuous diving [1, 2]. While similar fluctuations characterize pathophysiological events in non-hypoxia adapted species [45, 46], the cellular and molecular mechanisms which confer hypoxia tolerance in seals remain unclear. Here, we developed a proliferative arterial endothelial cell culture system using cells derived from elephant seals to identify key transcriptional regulators of the response to hypoxia. Our results show blunted angiogenic signaling despite rapid and sustained HIF-1α stabilization in seal cells. Moreover, seal cells also increase GSH pools during hypoxia, which likely help mitigate oxidant stress upon reoxygenation despite requiring energetic input under oxygen-limited conditions. High maximal and spare respiratory capacity at baseline and after hypoxia/reoxygenation and robust mitochondrial networks in seal cells may support such demands. Together, these data suggest that seal endothelial cells adapt to to meet oxygen availability during hypoxia rather than attempting to increase oxygen delivery in the face of limited supply.

### Canonical hypoxia signaling is decoupled from angiogenesis in seal cells

HIF-1 is the “master regulator” of the canonical response to hypoxia [47, 48], typically promoting both angiogenesis and glycolysis. In vivo, in vitro, and in silico analyses of HIF-1α structure and function indicate that marine mammal HIF-1α stabilization is highly sensitive to hypoxia [49–53]. In our experiments, HIF-1α stabilization occurred rapidly in seal endothelial cells exposed to hypoxia, and high levels of the protein persisted in seal cells through a 6-h exposure. Interestingly, seal cell expression of several HIF-1 targets implicated in vascular homeostasis (ANGPT2, MMP14, SLC9A1/NHE1) [54–57] remained stable during long-term hypoxia exposure in seal cells, while expression of these genes was variable or increased in human cells. Global transcriptional patterns supported this regulation as DE genes in seal cells predicted factors that regulate cell proliferation (FOXK1, ZBTB7A) and suppress endothelial activation (KLF11) [32, 58], while human cells show a consistently pro-angiogenic signal despite slower HIF-1α stabilization. Accordingly, hypoxia exposure increased gap closure rates in human but not seal cells. These results suggest that seal cells rapidly stabilize HIF-1α but further regulate the expression of putative HIF-1 targets during hypoxia exposure.

### Seal cells dampen inflammatory signaling throughout hypoxia exposure

Our data suggest continued regulation of inflammatory signaling in seal endothelial cells across 6 h of hypoxia exposure. Gene expression signatures early (≤ 1 h) in hypoxia exposure highlight downregulation of the TNF, NF-ĸB, and TGF-β pathways in seal cells. In contrast, enrichments in human cell gene expression identified substantially fewer inflammatory pathways. Furthermore, continued overrepresentation of TGF-β-related signaling pathways (including SMAD activity) coupled with increased expression of negative regulators including the dual specificity protein phosphatase MTMR4 and ubiquitin ligase NEDD4L in seal cells at 6 h, along with ZBTB7A at 30 min suggests that tight regulation of the TGF-β signaling cascade is critical in the seal cell response to hypoxia. Our data also suggest that altered NF-ĸB signaling during hypoxia exposure in seal cells may be regulated by TNF. NF-ĸB activation may be either pro-inflammatory or pro-survival depending on cellular context. The activity of both NF-ĸB and its inhibitor IKKβ are required for hypoxic HIF-1α accumulation and HIF-1 target gene expression [59]. Knockdown of the NF-ĸB subunit RelA in endothelial cells impairs smooth muscle cell proliferation [60] and NF-ĸB activity is implicated in eNOS downregulation during chronic intermittent hypoxia [61]. In Weddell seals, nitric oxide sensitivity varies across tissues and vessel beds but is generally blunted when compared to sheep [15], presumably to avoid hypoxic vasodilation during diving. Here, we found that tight regulation of NF-ĸB expression in seal cells exposed to hypoxia may promote cell survival and modulate vasodilatory signaling in the endothelium. These results corroborate a generally reduced inflammatory response in marine mammal whole blood stimulated with lipopolysaccharide [17, 62]. In contrast, changes to inflammatory signaling pathways occurring in human cells during short-term hypoxia exposure are supplanted by broad changes in transcriptional regulation during long-term exposure.

### Hypoxia promotes GSH synthesis in seal cells

High tissue and circulating GSH levels, as well as high activity of many glutathione-dependent antioxidant enzymes, including glutathione peroxidases (GPX) and glutathione *S*-transferases (GST) [18, 20–22, 63], suggest a critical role for GSH in protecting diving mammals against oxidative stress. Moreover, recent genomic work identified that several genes in the GSH metabolism pathway are under positive selection in marine mammals, including those enriched in our transcriptomic analyses [24–26, 64]. To date, however, the only GSH measurement during diving is in Weddell seals, with the observation that near-depletion of circulating GSH at the halfway point of restrained dives was followed by an increase above baseline levels within ~ 5 min of recovery [63]. Interestingly, we found that elephant seal vascular endothelial cells continue GSH synthesis during long-term hypoxia exposure alongside rapid increases in the expression of several GSH biosynthesis and GSH-dependent antioxidant enzymes. Hypoxic GSH accumulation in seal cells likely counteracts oxidant generation upon reoxygenation, protecting cells against oxidative damage and cellular stress. These results track with existing in vivo, in vitro, and in silico evidence supporting GSH’s role in maintaining redox homeostasis during diving in marine mammals [18, 20–22, 25, 26, 63]. Additionally, data from non-diving hypoxia-tolerant species indicate that increases in GSH prior to oxidative stress (driven by preconditioning) protect against later tissue injury [65, 66], potentially justifying the energetic cost of producing GSH in oxygen-limited conditions. Repeated diving behavior and terrestrial apnea bouts likely contribute to a “preconditioning” effect, which protects seal cells against oxidative damage, though genomic data also support a role for evolutionary pressure in shaping GSH metabolism in both pinnipeds and cetaceans [24–26, 64]. Fasting during development in elephant seals (during which time pups develop terrestrial apneas) stimulates GSH biosynthesis [21], and similar increases in GSH and related antioxidant enzymes occur with post-natal maturation in the deep-diving hooded seal [18]. In humans, breath hold training apparently eliminates a dive-associated decrease in circulating GSH after static apnea, though no increase over baseline levels is observed [67]. Therefore, it is likely that both behavioral preconditioning and underlying genomic/evolutionary changes in GSH metabolism drive hypoxic GSH upregulation in elephant seal endothelial cells.

### Hypoxic succinate accumulation may promote mitochondrial function in seal cells

In addition to GSH biosynthesis, other components of the glutathione metabolism pathway enriched in seal cells included those related to polyamine synthesis (via ODC1, SRM, and SMS) which may further protect cells against oxidant generation upon reoxygenation [68, 69]. Polyamines regulate the mitochondrial permeability transition pore via calcium flux, thus modulating the activity of the pyruvate dehydrogenase complex and impacting mitochondrial respiration [70]. Hypoxic accumulation of succinate in seal cells may derive from the conversion of the polyamine putrescine to succinate and sustain oxidative phosphorylation during hypoxia exposure. While excessive succinate accumulation due to reversal of succinate dehydrogenase (SDH) activity during hypoxia drives superoxide production via complex I during reoxygenation [10], inhibition of SDH activity by the anti-inflammatory metabolite itaconate may promote mild succinate accumulation without associated superoxide generation [71]. Additionally, succinate competitively inhibits prolyl hydroxylases such as those responsible for HIF-1α hydroxylation [72], supporting increased HIF-1α sensitivity to hypoxia in seal cells. Early increases in the HIF-1 target phosphoglucomutase 1 (PGM1) in seal cells may regulate glucose metabolism during hypoxia, consistent with glycolytic repression via ZBTB7A transcriptional control [73, 74]. Human cells, in contrast, upregulate several mitochondrial electron transport chain subunit components during short-term hypoxia exposure, consistent with increased mitochondrial branch length after 1 h, but overall changes in mitochondrial respiration in human cells following hypoxia/reoxygenation are milder than those in seal cells and are accompanied by higher ECAR, suggesting that human but not seal cells rely on glycolysis to support continued respiration during hypoxia.

## Conclusions

In summary, we found that elephant seal endothelial cells tightly regulate inflammation and mitochondrial dynamics and blunt angiogenic signaling during hypoxia exposure. These changes occur in parallel with increased GSH synthesis, which likely protects seal cells against oxidant generation during reoxygenation. Conversely, human cells signal for angiogenesis and vascular remodeling early and consistently throughout hypoxia exposure; these signals may destabilize the vasculature and increase susceptibility to inflammation and oxidative damage during reoxygenation. Here we identified candidate pathways supporting endothelial cell hypoxia tolerance in a deep diving seal. Recent genomic evidence from pinnipeds and cetaceans suggests convergent modifications in GSH metabolism and HIF function in marine mammals. Further investigation via co-culture with vascular smooth muscle, peripheral blood mononuclear cells, or serum from varied diving vertebrate taxa is required to anchor these responses in the context of the sustained vasoconstriction characteristic of the diving response.

## Methods

### Tissue acquisition

Northern elephant seal *(Mirounga angustirostris)* placentae were obtained under NMFS permit # 19108 (PI: Daniel Costa, UC Santa Cruz). Whole placentae were collected after live births (< 1 h) from the beach at Año Nuevo Reserve (San Mateo, CA) during the pupping season. The generation of cell lines was conducted under NMFS permit # 22479. De-identified human placentae were donated following uncomplicated live births at Sutter Health’s California Pacific Medical Center. All placentae were maintained at 4°C until the time of dissection. All dissections were performed within 6 h of tissue collection.

### Primary cell isolation and culture

Arteries were dissected from placental tissue and rinsed with ice-cold sterile Hanks’ balanced salt solution (HBSS; Gibco, Waltham, MA, USA). Arteries were cut open lengthwise, flushed with HBSS, and placed in a collagenase type II solution (500 U/mL in DMEM; Worthington Biochemical, Lakewood, NJ, USA) for 30 min in a tissue culture incubator (37°C, 5% CO_2_, humidified). The collagenase solution was blocked with complete growth medium. The endothelial surface of the arteries was scraped into tissue culture-treated dishes containing complete growth medium and transferred to a tissue culture incubator. Endothelial cell islands were visible in the culture dishes after 24 h. The growth medium was changed daily for the first 4–5 days at passage 0. Seal cells were maintained in Dulbecco’s modified Eagle medium (DMEM; Gibco, catalog # 11885–084) supplemented with 10% fetal bovine serum (Seradigm, Avantor, Mexico), 10 mM HEPES (Gibco), 1% (1 ×) Antibiotic–Antimycotic (Gibco), and 4 ug/mL endothelial cell growth supplement (ECGS; Corning, Corning, NY, USA). Human cells were grown in commercial endothelial cell medium (Sciencell Research Laboratories, catalog # 1001, Carlsbad, CA, USA). Confluent cultures were cryopreserved at passage 1. Pooled stock cultures were created for human (*n* = 2–3) and seal (*n* = 3) cells to minimize the impacts of individual variation. Northern elephant seal trophoblasts were isolated by mincing placental labyrinth in a collagenase type II solution, followed by 5 min in a tissue culture incubator. The collagenase solution was blocked with complete medium and passed through a 100 µm filter. The filtrate was centrifuged at 200 × *g* for 5 min at 4°C. The pellet was washed once in HBSS and resuspended in trophoblast growth medium (Sciencell Research Laboratories, catalog # 7121), then plated on tissue culture-treated dishes coated with collagen IV. Given that primary cells can lose phenotypic features with increasing passage number, we conducted all experiments in cells between passages 4–7 (and species were passage-matched within experiments) to mitigate any such changes to the extent possible. All cells were switched to the same medium formulation the day prior to experiments.

### Cell characterization

The endothelial phenotype of the cell preparations was confirmed by DiI-acetylated LDL uptake (Invitrogen, catalog # L3484), RT-qPCR for vascular endothelial cadherin (CD144) and platelet endothelial cell adhesion molecule-1 (PECAM-1), and immunostaining for PECAM-1 (CD31; Novus Biologicals, catalog # NB100-2284, 1:100). The DiI-acetylated LDL uptake assay was conducted following manufacturer instructions. RT-qPCR was conducted using our previously published methods [75] with minor modifications in the reaction conditions: 1 min at 95°C followed by 40 cycles of 20 s at 95°C and 30 s at 60°C. Primer sequences are listed in Additional file 3: Table S5. Relative mRNA expression (fold change) was calculated as 2^−ΔΔCt^. Immunostaining was conducted following the protocol described in [76]. Tube formation was assessed by plating 10,000 cells per well in a µ-slide 3D angiogenesis glass bottom slide (Ibidi, Fitchburg, WI, USA, catalog # 81506) coated with Matrigel. All imaging was conducted using a Zeiss Axio Observer 7 inverted microscope fitted with 10 × and 20 × objectives and Zen software.

### Extracellular flux assays

Oxygen consumption was measured using the Seahorse Mitochondrial Stress Test Kit (Agilent Technologies, Santa Clara, CA, USA) and an XFp Extracellular Flux Analyzer; 30,000 cells/well were seeded in fibronectin-coated miniplates. Cells were washed with serum-free assay medium (Seahorse XF DMEM pH 7.4, 5.56 mM glucose, 1 mM pyruvate, 4 mM L-glutamine) and incubated at 37°C in a non-CO_2_ incubator for 1 h before assay. Oxygen consumption rates (OCR) were measured in the presence of oligomycin (1 µM), carbonyl cyanide-*p*-trifluoromethoxyphenylhydrazone (FCCP; 1 µM for human cells, 2 µM for seal cells), and rotenone/antimycin A (0.5 µM). Optimal FCCP concentrations were determined empirically for each species. Protein content was measured in each well at the end of the assay using the Qubit Protein Assay Kit (Molecular Probes, Eugene, OR, USA). Oxygen consumption rate (OCR) and extracellular acidification rate (ECAR) were normalized to protein content. Mitochondrial function was calculated according to [77] and normalized to basal respiration per well.

### Hypoxia exposure

Cells were incubated in an InVivO_2_ physiological cell culture workstation (Baker Ruskinn, Sanford, Maine, USA) under the following conditions: 1% O_2_, 5% CO_2_, 37°C for 15 min, 30 min, 1 h, 2 h, 4 h, and 6 h. Protein and RNA were collected within the hypoxic environment. Control cells remained in a standard tissue culture incubator (21% O_2_, 37°C, 5% CO_2_). Cell viability was confirmed in cells undergoing hypoxia exposure using a commercial viability/cytotoxicity kit (Thermo Fisher, catalog number: R37601). In some experiments, extracellular flux assays were conducted after hypoxia/reoxygenation treatments consisting of 1 h at 0.5% O_2_ (37°C, 5% CO_2_) followed by 30 min in room air (37°C, no CO_2_) prior to assay.

### Immunoblotting

Western blot was conducted using our previously published methods [78]. Briefly, cells were scraped in DPBS (Gibco) containing 1% Triton X-100 and 2% Halt Protease and Phosphatase Inhibitor Cocktail (Thermo Scientific, Waltham, MA, USA). Lysates were sonicated and centrifuged. Total protein content in the supernatant was determined using a BCA Rapid Gold Protein Assay (Pierce, Rockford, IL, USA). Proteins were resolved using SDS-PAGE and transferred onto nitrocellulose membranes. Membranes were blocked with Odyssey blocking buffer (LICOR, Omaha, NE, USA) and incubated overnight with an antibody against hypoxia inducible factor-1α (HIF-1α, Cell Signaling Technology # 36169; RRID:AB_2799095; 1:500). Proteins were visualized using IRDye 800CW secondary antibodies (LICOR) and a two-color near-infrared system (Azure c500, Azure Biosystems, Dublin, CA, USA). Membranes were stripped and reprobed with an antibody against β-actin (Cell Signaling Technology # 4967; RRID:AB_330288; 1:2,000). The intensity of individual bands was quantified using FIJI v2.1.0 and normalized to β-actin.

### Biochemical assays and live-cell imaging during hypoxic conditions

Glutathione content was measured under normoxic conditions and after exposure to 30 min and 6 h hypoxia using the GSH-Glo Assay Kit (Promega, Madison, WI, USA) according to manufacturer instructions. Luminescence was measured using a SpectraMax M3 Microplate Reader (Molecular Devices, San Jose, CA, USA). Changes in GSH levels were also measured in hypoxic live cells using ThiolTracker Violet (Invitrogen, catalog # T10096). Cells were loaded with 20 µM ThiolTracker Violet glutathione detection reagent and 100 nM SYTO 16 Green Fluorescent Nucleic Acid Stain (Invitrogen, catalog # S7578) and imaged within the hypoxic environment using an LS620 microscope (Etaluma, Carlsbad, CA, USA) fitted with a 20 × objective at 0, 30 min, and 6 h at 1% O_2_. Fluorescence intensity was quantified in 12 random cells per field using FIJI v2.1.0.

Intracellular succinate concentration was measured in cells under normoxic conditions and after exposure to 1- and 6-h hypoxia using a commercial assay (Sigma catalog # MAK184, St. Louis, MO, USA) according to manufacturer instructions. Absorbance at 450 nm was measured using a SpectraMax M3 Microplate Reader (Molecular Devices, San Jose, CA, USA). Succinate concentrations per well were normalized to protein content using a Pierce Rapid Gold Protein Assay (Pierce, Rockford, IL, USA).

### Cell migration assay

Cells were plated in an Ibidi 2-well silicone culture insert (Ibidi, catalog # 81176) adhered to a TC-treated 35-mm glass bottom dish and grown to confluence. At confluence, culture inserts were removed to create a “scratch.” Dishes were washed with DPBS to remove unattached cells, and the media was changed. Cells were incubated in control (normoxia) or hypoxia (1% O_2_) conditions for 6 h. Live cells were imaged using a Zeiss Axio Observer 7 inverted microscope fitted with a 10 × objective at 1, 2, 4, and 6 h. Cell migration was calculated as the percent difference in the cell-free area in hypoxic dishes compared to control at each time point using the ScratchAssayAnalyzer function in the MiToBo FIJI plugin.

### Mitochondrial staining and network analysis via confocal microscopy

Live cells were stained with MitoTracker Red CMXRos (Cell Signaling Technology, Danvers, MA) and NucBlue (Hoescht 33,342, Invitrogen) under normoxic conditions and after 1- and 6-h hypoxia (1% O_2_) followed by 30 min reoxygenation. Cells were fixed in ice-cold 1:1 methanol:acetone, rinsed with PBS, and imaged in VECTASHIELD mounting media (Vector Laboratories, Burlingame, CA) using a Zeiss LSM 710 microscope fitted with a 63 × objective (Plan-Apochromat 1.4 NA oil, 0.13 mm) and Zen software. Mitochondrial footprint and mean branch length were determined from unfiltered binarized images using the MiNA (v2.0) FIJI plugin (https://github.com/StuartLab/MiNA).

### RNAseq

Hypoxic cells were lysed in Buffer RLT without β-mercaptoethanol (Qiagen, Germantown, MD, USA). RNA extractions were carried out using an RNeasy Mini Kit (Qiagen) including on-column DNase treatment according to manufacturer instructions. DNase treatment was confirmed by a lack of genomic amplification using PCR. RNA yield was quantified using a Nanodrop 1000 Spectrophotometer (Thermo Scientific). RNA integrity number (RIN) was determined using an Agilent 2100 Bioanalyzer kit for total eukaryotic RNA (Agilent Technologies, Santa Clara, CA, USA). Samples underwent library preparation and sequencing at the UC Berkeley Functional Genomics and Vincent J. Coates Genomics Sequencing Laboratories. A KAPA RNA HyperPrep Kit (catalog # KK8541; Roche, Basel, Switzerland) was used to prepare cDNA libraries from poly(A)-captured mRNA with TruSeq adapters. Three replicates per condition were used to generate libraries, with a sequencing depth of 25 M reads per sample on a NovaSeq platform (Illumina, San Diego, CA, USA).

### Transcriptome analyses

The elephant seal *(Mirounga angustirostris)* genome (https://www.dnazoo.org/assemblies/Mirounga_angustirostris) was annotated as described in our previous work [75]. Reads were mapped to the elephant seal or human genomes (version GRCh38) using STAR aligner [79]. Transcript levels were quantified with RSEM v1.3.1 [80]. Expression levels were converted to *z*-scores and normalized by the mean and variance across all replicates and conditions for each gene. The median *z*-score was selected for each gene and used to construct a Euclidean distance matrix. Genes that changed in a concerted manner across hypoxia exposure times were clustered by *k*-means. Optimal clustering was selected at *k* = 6 due to diminishing returns at higher *k*. Within each cluster, GSEA with KEGG terms was used to identify enriched biological pathways; enrichments were not assessed for “no change” clusters (seal: cluster 4; human: cluster 2). Additionally, genes differentially expressed between control and each timepoint were identified using EBSeq with a false discovery rate of 5% [81]. GSEA for Reactome pathways was conducted using a summary list of genes differentially expressed at 15 min, 30 min, and 1 h versus control [82]. Functional Interaction Networks (FIN) were built for the same gene lists using Cytoscape v3.8.2 [83, 84]. *Cis*-regulatory analyses were conducted using iRegulon in Cytoscape [85].

### Statistical analyses

Statistical analyses were performed using GraphPad Prism v9.3.1. Normality was evaluated using D’Agostino–Pearson or Shapiro–Wilk tests, depending on sample size. When normality assumptions were met, differences between groups with equal variance were assessed by independent two-tailed *t-*test or one-way ANOVA. When homoscedasticity assumptions were not met, differences between groups were determined by two-tailed *t-*test with Welch’s correction or Welch’s ANOVA. In cases where data were not normally distributed, Kruskal–Wallis tests were used. In all cases, we corrected for multiple comparisons by setting FDR to 0.05; post hoc analyses used the two-stage linear step-up procedure of Benjamini et al. [86]. Data are presented as mean ± s.e.m.

### Supplementary Information

**Additional file 1: Supplementary Figures S1-S6. Figure S1.** **Representative images for mitochondrial staining after 0, 1, or 6 h hypoxia exposure followed by 30 min reoxygenation. **Upper: seal. Lower: human. MitoTracker Red CMXRos (red). Hoescht (blue). Scale bar is 50 µm. **Figure S2. Uncropped western blots. **(A) HIF-1α. (B) β-actin. **Figure S3. Raw gap area for all species and conditions during scratch assay. Figure S4. Total within cluster sum of squares for clustering results from *k*=1...30).** Upper: seal. Lower: human. Diminishing returns were observed after 6 clusters thus *k*=6 (pink) was selected as the optimal clustering. **Figure S5.** ***k-*means clustering and KEGG pathway enrichment for human cells.** *k*-means clustering of gene expression data for human cells exposed to 1% O_2_ for up to 6 h. (B) KEGG pathway enrichment for human clusters. Numbers on the right axis correspond to human cluster numbers. **Figure S6.** **Long-term hypoxia exposure modulates transcription and translation in human cells. **(A) Reactome pathway enrichments for genes DE at all late time points in human cells; no pathways were enriched in seal. (B) GSEA for genes DE at 6 h versus control in human cells; no pathways were enriched in seal. Normalized enriched score <0 indicates net downregulation of the pathway.

**Additional file 2:** **Supplementary tables S1-S4. Table S1. **Percent live cells after hypoxia exposure. **Table S2.** Fold change in expression of respiratory electron chain components in response to short-term hypoxia exposure in human cells. **Table S3.** Fold change in expression of respiratory electron chain components in response to long-term hypoxia exposure in human cells. **Table S4.** Fold change in expression of TGF-β signaling components in response to 6 h exposure in seal cells.

**Additional file 3:** **Supplementary table S5.**

## Data Availability

All data generated or analyzed during this study are included in this published article, its supplementary information files, and publicly available repositories. Raw RNAseq data are available at NCBI’s Sequence Read Archive BioProjects PRJNA1017894 [87] and PRJNA1017895 [88]. Code and results are available on Dryad at 10.5061/dryad.7pvmcvf0p [89].

## Abbreviations

HIF: Hypoxia inducible factor
GSH: Glutathione
Ac-LDL: Acetylated low-density lipoprotein
GSEA: Gene set enrichment analysis
DE: Differentially expressed (genes)
