# Supplementary material for: Hypoxia exposure blunts angiogenic signaling and upregulates the antioxidant system in endothelial cells derived from elephant seals
Source: BMC Biol. 2024 Apr 23;22:91. doi: 10.1186/s12915-024-01892-3 (PMC11040891; doi:10.1186/s12915-024-01892-3)
Supplement: Supplementary file 1 — Additional file 1: Supplementary Figures S1-S6. Figure S1. Representative images for mitochondrial staining after 0, 1, or 6 h hypoxia exposure followed by 30 min reoxygenation. Upper: seal. Lower: human. MitoTracker Red CMXRos (red). Hoescht (blue). Scale bar is 50 µm. Figure S2. Uncropped western blots. (A) HIF-1α. (B) β-actin. Figure S3. Raw gap area for all species and conditions during scratch assay. Figure S4. Total within cluster sum of squares for clustering results from k=1...30). Upper: seal. Lower: human. Diminishing returns were observed after 6 clusters thus k=6 (pink) was selected as the optimal clustering. Figure S5. k-means clustering and KEGG pathway enrichment for human cells. k-means clustering of gene expression data for human cells exposed to 1% O2 for up to 6 h. (B) KEGG pathway enrichment for human clusters. Numbers on the right axis correspond to human cluster numbers. Figure S6. Long-term hypoxia exposure modulates transcription and translation in human cells. (A) Reactome pathway enrichments for genes DE at all late time points in human cells; no pathways were enriched in seal. (B) GSEA for genes DE at 6 h versus control in human cells; no pathways were enriched in seal. Normalized enriched score <0 indicates net downregulation of the pathway. [file 12915_2024_1892_MOESM1_ESM.pdf]

Figure S1.

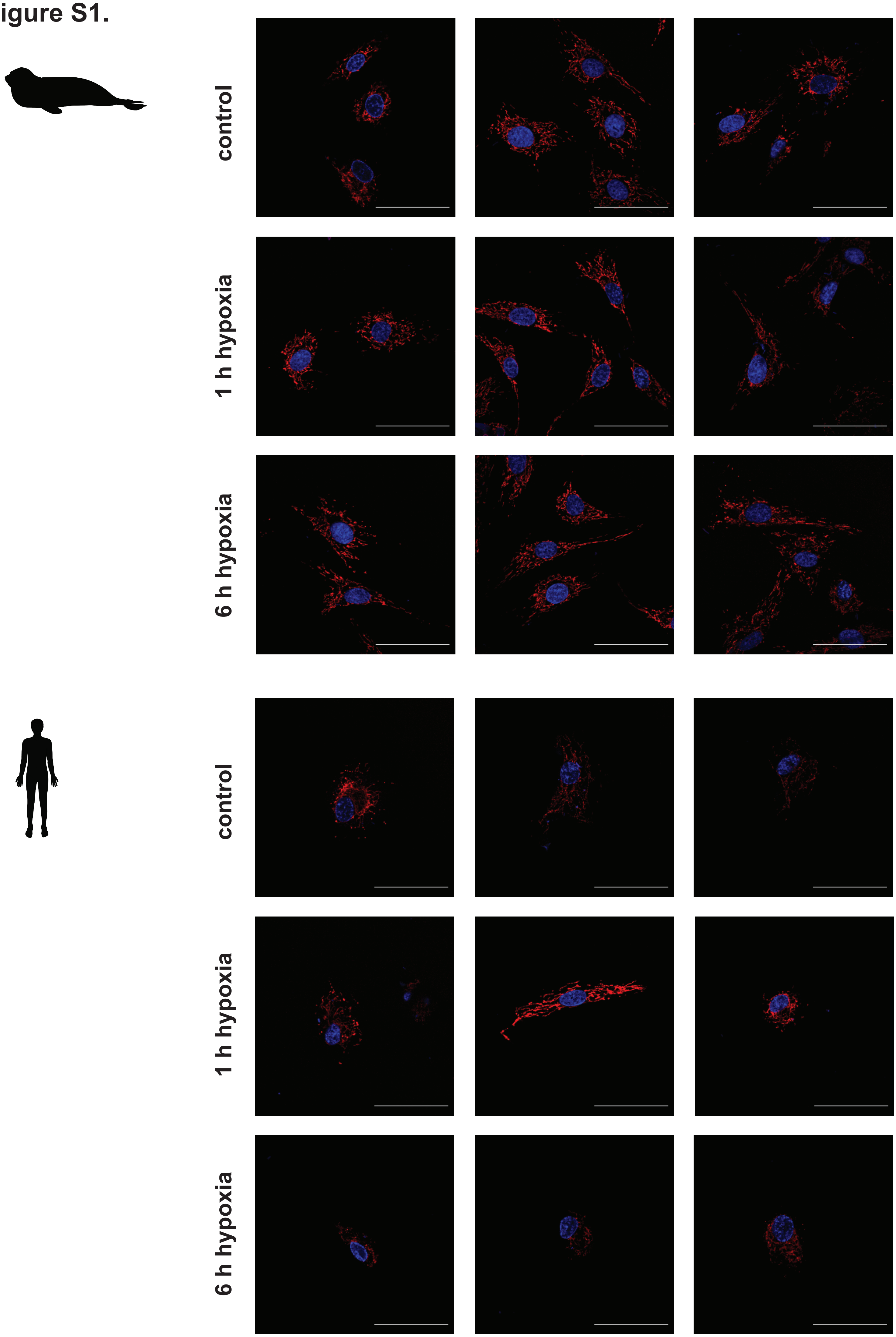
control
1 h hypoxia
6 h hypoxia
control
1 h hypoxia
6 h hypoxia

Figure S2.

A

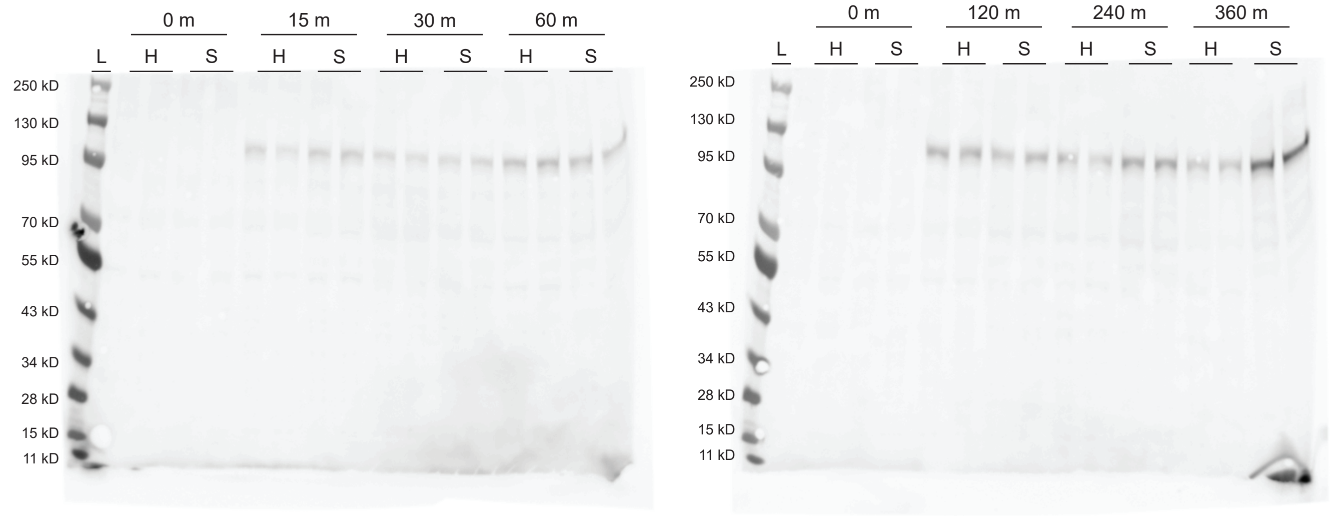
0 m
15 m
30 m
60 m
L
H
S
250 kD
130 kD
95 kD
70 kD
55 kD
43 kD
34 kD
28 kD
15 kD
11 kD
120 m
240 m
360 m

B

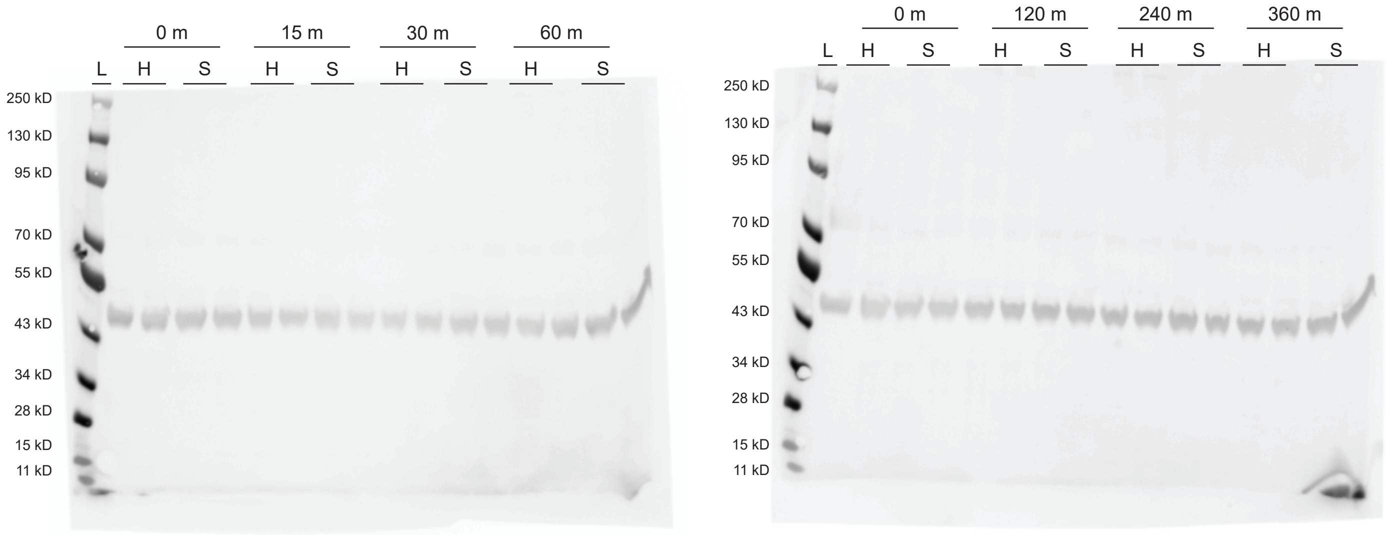
0 m
15 m
30 m
60 m
L
H
S
250 kD
130 kD
95 kD
70 kD
55 kD
43 kD
34 kD
28 kD
15 kD
11 kD
120 m
240 m
360 m

Figure S3.

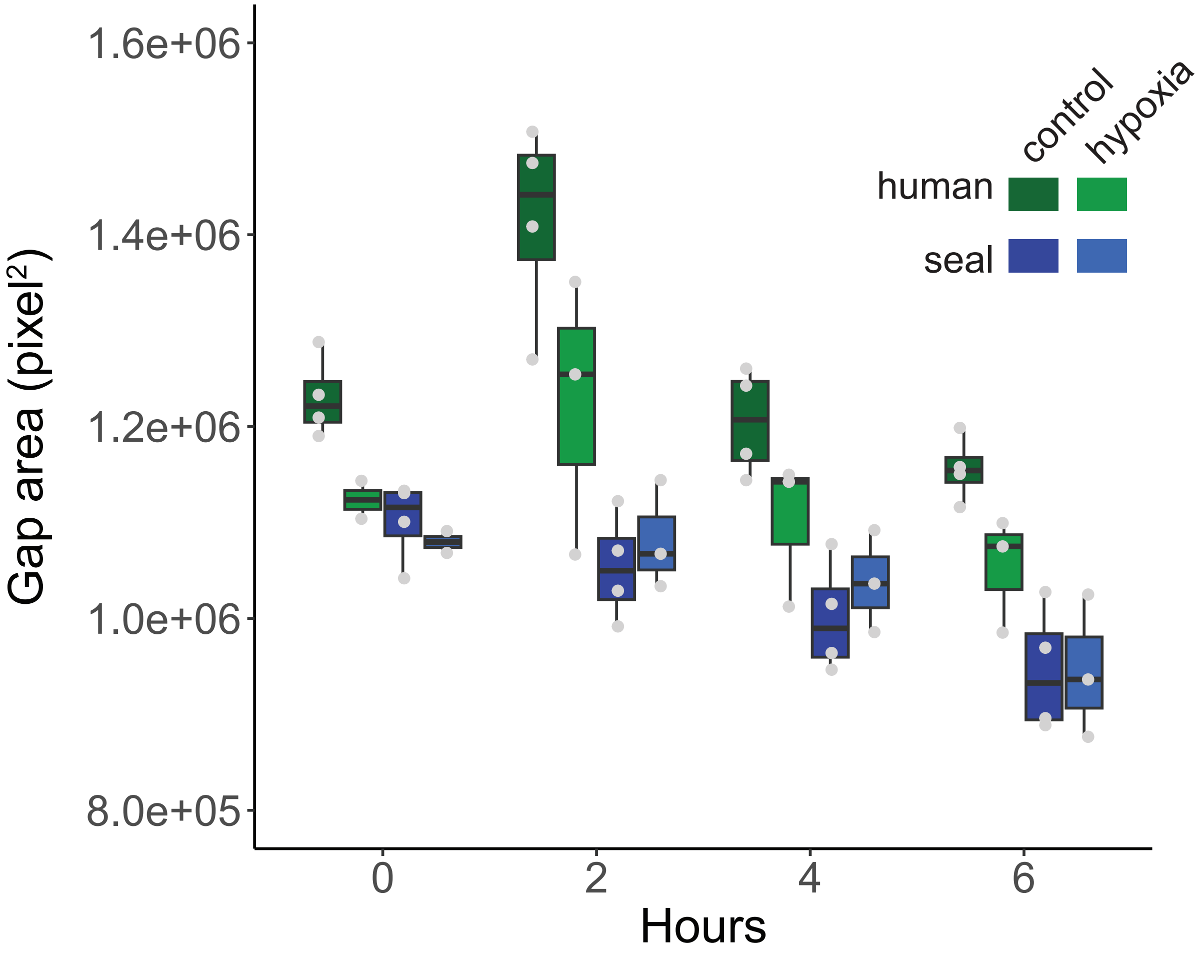

1.6e+06
1.4e+06
1.2e+06
1.0e+06
8.0e+05
Gap area (pixel²)
0
2
4
6
Hours
control
hypoxia
human
seal

Figure S4.

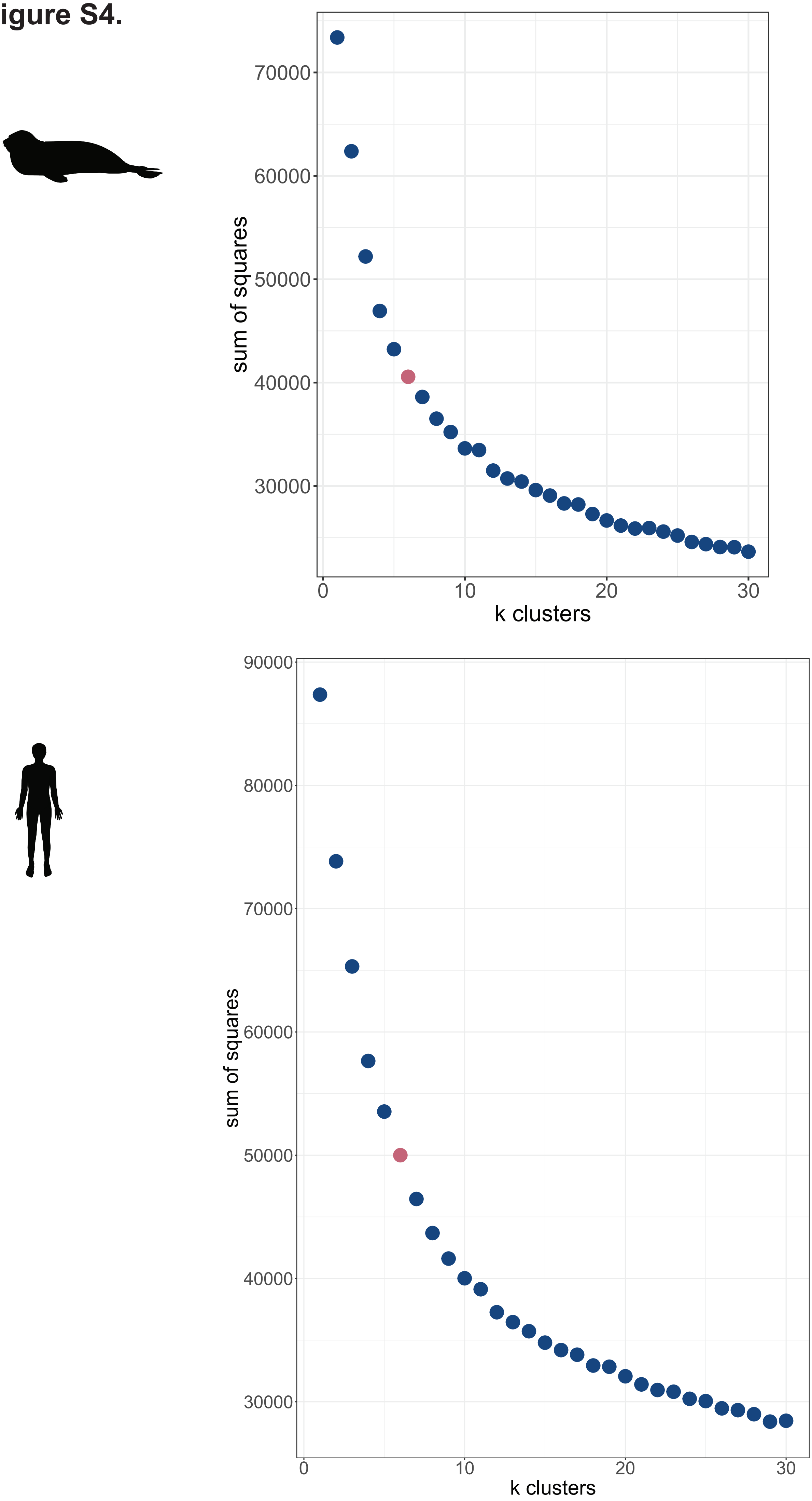
sum of squares
70000
60000
50000
40000
30000
0
10
20
30
k clusters
sum of squares
90000
80000
70000
60000
50000
40000
30000
0
10
20
30
k clusters

Figure S5.

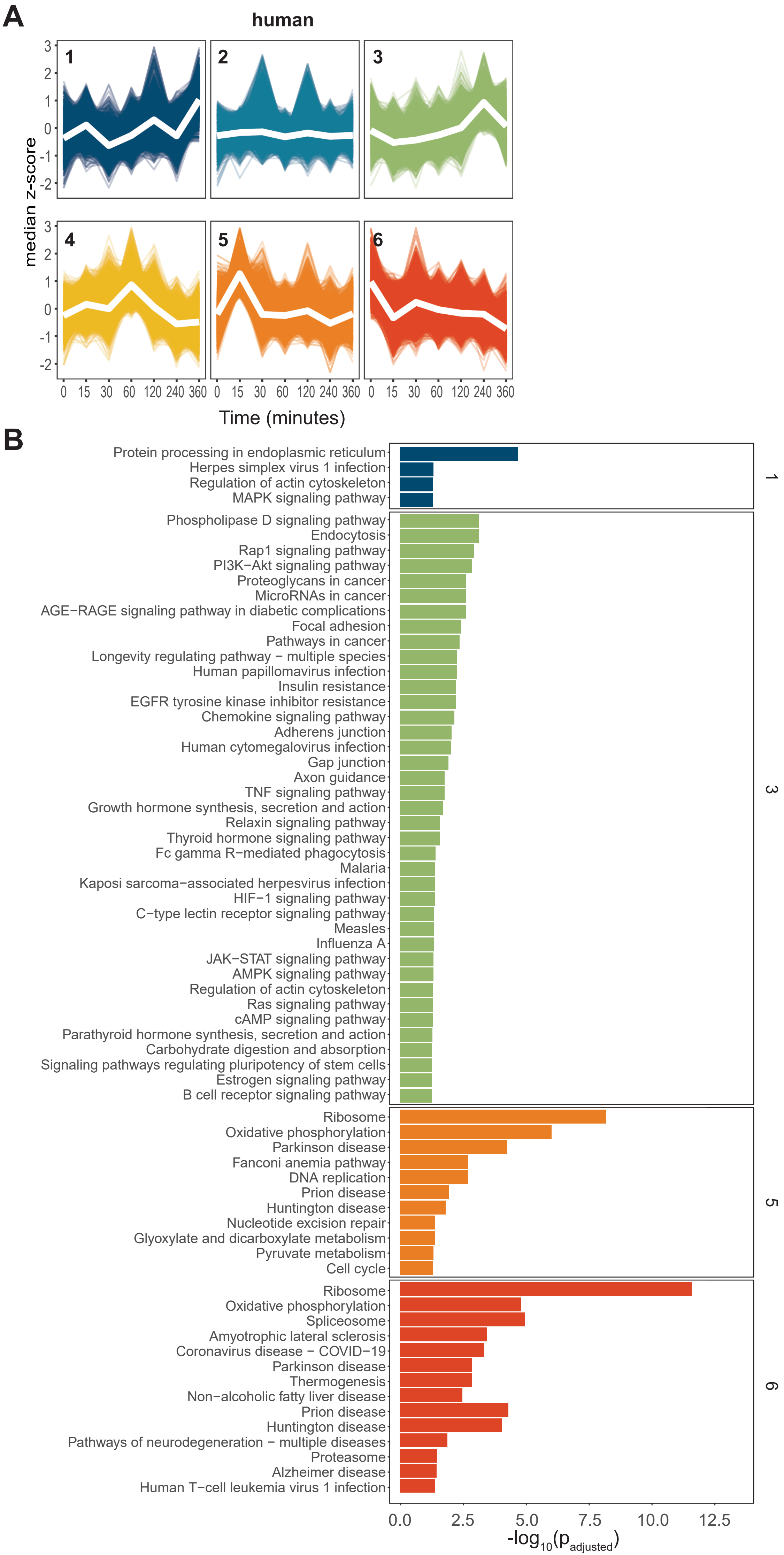
A
human
1
2
3
4
5
6
median z-score
3
2
1
0
-1
-2
0 15 30 60 120 240 360
Time (minutes)
B
Protein processing in endoplasmic reticulum
Herpes simplex virus 1 infection
Regulation of actin cytoskeleton
MAPK signaling pathway
Phospholipase D signaling pathway
Endocytosis
Rap1 signaling pathway
PI3K−Akt signaling pathway
Proteoglycans in cancer
MicroRNAs in cancer
AGE−RAGE signaling pathway in diabetic complications
Focal adhesion
Pathways in cancer
Longevity regulating pathway − multiple species
Human papillomavirus infection
Insulin resistance
EGFR tyrosine kinase inhibitor resistance
Chemokine signaling pathway
Adherens junction
Human cytomegalovirus infection
Gap junction
Axon guidance
TNF signaling pathway
Growth hormone synthesis, secretion and action
Relaxin signaling pathway
Thyroid hormone signaling pathway
Fc gamma R−mediated phagocytosis
Malaria
Kaposi sarcoma−associated herpesvirus infection
HIF−1 signaling pathway
C−type lectin receptor signaling pathway
Measles
Influenza A
JAK−STAT signaling pathway
AMPK signaling pathway
Regulation of actin cytoskeleton
Ras signaling pathway
cAMP signaling pathway
Parathyroid hormone synthesis, secretion and action
Carbohydrate digestion and absorption
Signaling pathways regulating pluripotency of stem cells
Estrogen signaling pathway
B cell receptor signaling pathway
Ribosome
Oxidative phosphorylation
Parkinson disease
Fanconi anemia pathway
DNA replication
Prion disease
Huntington disease
Nucleotide excision repair
Glyoxylate and dicarboxylate metabolism
Pyruvate metabolism
Cell cycle
Ribosome
Oxidative phosphorylation
Spliceosome
Amyotrophic lateral sclerosis
Coronavirus disease − COVID−19
Parkinson disease
Thermogenesis
Non−alcoholic fatty liver disease
Prion disease
Huntington disease
Pathways of neurodegeneration − multiple diseases
Proteasome
Alzheimer disease
Human T−cell leukemia virus 1 infection
1
3
5
6
0.0 2.5 5.0 7.5 10.0 12.5
-log10(padjusted)

Figure S6.

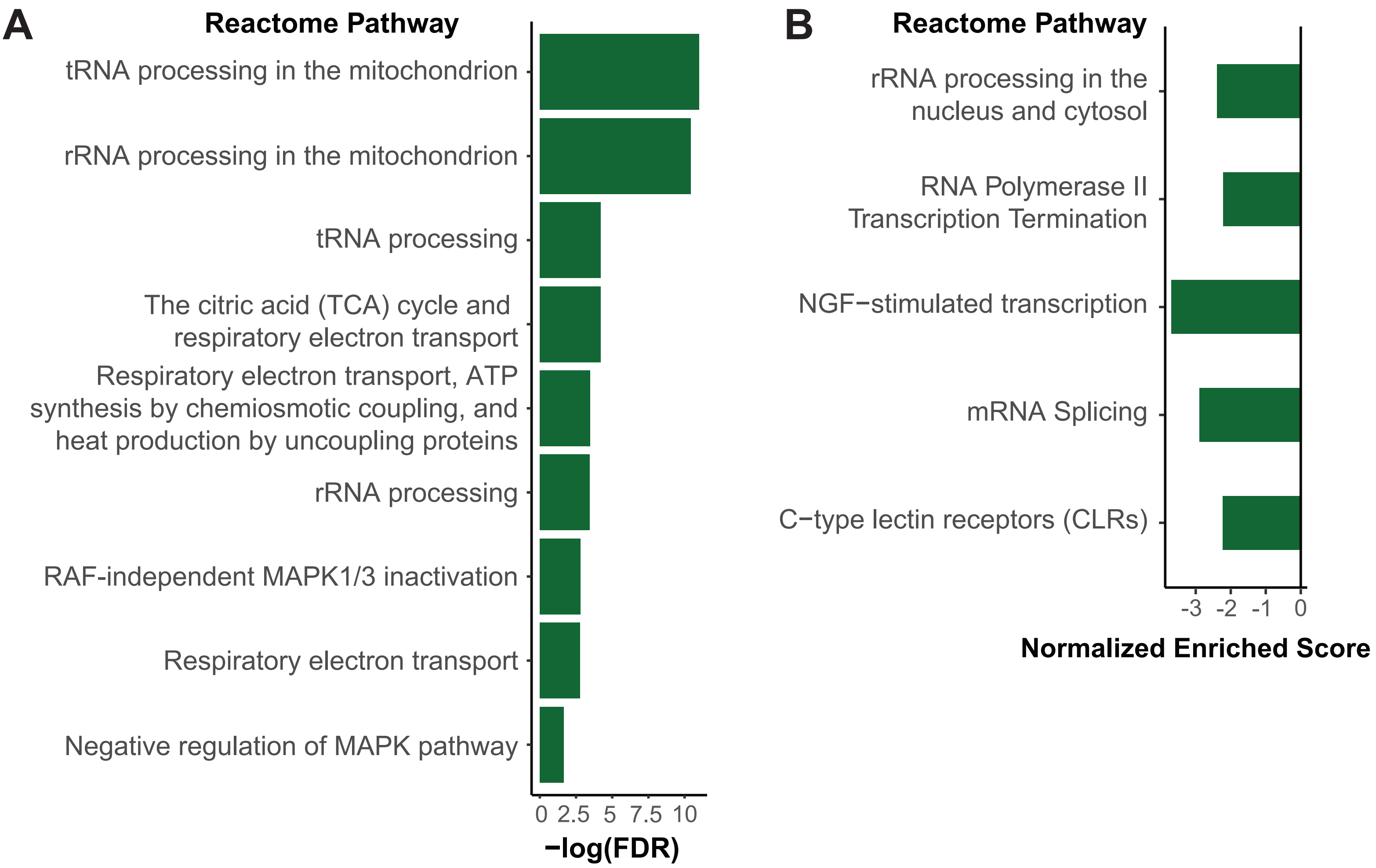
A
Reactome Pathway
tRNA processing in the mitochondrion
rRNA processing in the mitochondrion
tRNA processing
The citric acid (TCA) cycle and respiratory electron transport
Respiratory electron transport, ATP synthesis by chemiosmotic coupling, and heat production by uncoupling proteins
rRNA processing
RAF-independent MAPK1/3 inactivation
Respiratory electron transport
Negative regulation of MAPK pathway
0
2.5
5
7.5
10
−log(FDR)
B
Reactome Pathway
rRNA processing in the nucleus and cytosol
RNA Polymerase II Transcription Termination
NGF−stimulated transcription
mRNA Splicing
C−type lectin receptors (CLRs)
-3
-2
-1
0
Normalized Enriched Score
