# Supplementary material for: Hypoxia exposure blunts angiogenic signaling and upregulates the antioxidant system in endothelial cells derived from elephant seals
Source: BMC Biol. 2024 Apr 23;22:91. doi: 10.1186/s12915-024-01892-3 (PMC11040891; doi:10.1186/s12915-024-01892-3)
Supplement: Supplementary file 2 — Additional file 2: Supplementary tables S1-S4. Table S1. Percent live cells after hypoxia exposure. Table S2. Fold change in expression of respiratory electron chain components in response to short-term hypoxia exposure in human cells. Table S3. Fold change in expression of respiratory electron chain components in response to long-term hypoxia exposure in human cells. Table S4. Fold change in expression of TGF-β signaling components in response to 6 h exposure in seal cells. [file 12915_2024_1892_MOESM2_ESM.pdf]

Table S1. Percent live cells. Data are mean ± s.e.m. for 4-5 images per dish, with n=3 dishes per species per time. No statistically significant differences were detected between time points within each species.

	Control	30 min hypoxia	6 h hypoxia
Human	99.41 ± 0.34 %	99.05 ± 0.62 %	99.30 ± 0.36 %
Elephant seal	99.79 ± 0.05 %	99.90 ± 0.05 %	99.90 ± 0.05 %

Table S2. Fold change in expression of respiratory electron chain components in response to short-term hypoxia exposure in human cells. Fold change is expressed as a comparison to species baseline.

Gene name	15 min	30 min	60 min
mt-cyb	1.46	1.63	1.59
mt-atp8	1.96	1.82	1.88
mt-co1	1.90	1.76	1.74
mt-co2	1.67	1.77	1.70
mt-nd1	1.53	1.85	1.68
mt-nd2	1.62	1.80	1.64
mt-nd4	1.93	2.11	2.08
mt-nd5	1.57	1.77	1.80
mt-nd6	1.66	1.78	1.74

Table S3. Fold change in expression of respiratory electron chain components in response to long-term hypoxia exposure in human cells. Fold change is expressed as a comparison to species baseline.

Gene name	120 min	240 min	360 min
dlat	1.15	1.23	1.18
ldha	1.34	1.66	2.28
mt-atp6	1.67	1.84	1.62
mt-atp8	1.99	1.84	1.97
mt-co1	1.87	1.83	2.08
mt-co2	1.64	1.61	1.69
mt-co3	1.86	1.84	1.90
mt-cyb	1.67	1.75	1.58
mt-nd1	1.70	1.80	1.53
mt-nd2	1.68	1.72	1.52
mt-nd4	2.14	2.38	2.27
mt-nd5	1.79	2.02	2.00
mt-nd6	1.75	2.03	2.01
pdk1	2.24	3.27	4.60

Table S4. Fold change in expression of TGF-β signaling components in response to 6 h exposure in seal cells. Fold change is expressed as a comparison to species baseline.

Gene name	Fold change
mtmr4	2.48
neddl1	1.65
serpine1	1.38
tgfbr1	1.36
smad2	1.28
itgb1	1.13
hdac1	0.81
smurf1	0.76
f11r	0.71
smurf2	0.66
e2f4	0.62
tgfbr2	0.58
smad3	0.55
ski	0.54
skil	0.34
tgif1	0.28

junb	0.27
smad7	0.23
