# Supplementary material for: Hypoxia exposure blunts angiogenic signaling and upregulates the antioxidant system in endothelial cells derived from elephant seals
Source: BMC Biol. 2024 Apr 23;22:91. doi: 10.1186/s12915-024-01892-3 (PMC11040891; doi:10.1186/s12915-024-01892-3)
Supplement: Supplementary file 3 — Additional file 3: Supplementary table S5. [file 12915_2024_1892_MOESM3_ESM.pdf]

Table S5. Primer sequences.

Target gene	Sequence 5' to 3'
ve-cadherin (cd144)	CCC AGA ACC GGA TGA CCA AG
	TTT CGG ATG GAG ACG CTG CT
pecam-31 (cd31)	CAA TAG AAG GCG GGG TCG TG
	CGT GGC TTG GCA CTG GAA GT
actin	CGG TCA GTT CAT GGC TGA GG
	AAG GCT CGG ACC TTC CCA AC
gapdh	CAA GGC TGA GAA CGG GAA GC
	ATC GGC AGA GGG AGC AGA GA
